# Who Is Attending? The Role of Child Ethnicity and Maternal Demographics in Research Engagement and Early Identification of Autism

**DOI:** 10.3390/brainsci13060903

**Published:** 2023-06-02

**Authors:** Ifrah Abdullahi, Nancy Sadka, Melissa Gilbert, Josephine Barbaro

**Affiliations:** 1 Olga Tennison Autism Research Centre, School of Psychology and Public Health, La Trobe University, Bundoora, VIC 3086, Australiam.gilbert@latrobe.edu.au (M.G.);; 2 Cooperative Research Centre for Living with Autism (Autism CRC), Indooroopilly, QLD 4068, Australia

**Keywords:** developmental surveillance, autism, child, ethnicity

## Abstract

Inequitable access to early autism developmental surveillance is evident globally. However, there is limited research examining autism diagnosis, ethnicity, and community profiles when engaging in research for the early identification of autism. We aimed to understand the relationships between child ethnicity, maternal demographics, and autism diagnosis, comparing retrospective data from the 2016 census for eight local government areas (LGAs) in Victoria, Australia. Maternal and child health (MCH) nurses monitored 13,511 children under 42 months for the early signs of autism using the Social Attention Communication Surveillance-Revised (SACS-R) and SACS-R Preschool (SACS-PR) tools during well-child checks. Of these, 340 children with a “high likelihood” of autism attended developmental assessments. Participants’ maternal ethnicity (‘European maternal ethnicity’, EME; ‘non-European maternal ethnicity,’ N-EME; ‘mixed maternal ethnicity,’ MME’), socioeconomic factors, and autism prevalence were compared to their LGA community. Results indicated that study participants were representative of their LGA communities, though bi- and multilingualism was higher in our cohort. Differences in current maternal employment, maternal education, annual family income, and autism prevalence were found between the N-EME, EME, and MME groups. Our study found that research engagement was driven by maternal education, maternal employment, and annual family income, and further research is required to understand these relationships.

## 1. Introduction

Autism is characterized by differences in social interaction, communication, and the presence of sensory and repetitive behaviours and special interests [1]. The prevalence of autism and related disabilities varies globally, with estimates of 1 in 36 in the United States of America (US) [2,3], 1 in 70 in the United Kingdom (UK) [4], and 1 in 70–80 in Australia [5,6,7]. In a recent study in Australia, the estimated autism prevalence was 1 in 31 children under four in Victoria [8].

Internationally, a significant increase in autism prevalence has been reported over the last two to three decades with many reasons suggested, including broadening diagnostic criteria, more thorough identification and diagnosis, or the possibility of an actual increase in prevalence [9,10,11,12]. Through the Autism and Developmental Disabilities Monitoring (ADDM) Network in the US, a difference in prevalence has also been observed from 2000 to 2016 in children from non-European ethnic backgrounds compared to European-American children [13,14,15,16]. A 2020 US study found no statistically significant difference in autism prevalence between African American and European-American children aged eight years, a first in ADDM network’s history, as this decline indicates potential current improvements in early and equitable identification of autism in the US [17]. However, disparities for children of colour persisted and were evident in age at diagnosis; African American children still had, on average, a later median age at autism diagnosis than Hispanic and European-American children (48 months compared to 44 and 42 months, respectively), and African American children diagnosed with co-occurring intellectual disability (ID) were still less likely to receive a timely autism diagnosis [17]. Furthermore, in the most recent edition of the ADDM network’s community autism report, there was a re-emergence of prevalence differences, with higher identification of autistic 8-year-old African American, Hispanic, and Asian/Pacific Islander children compared to 8-year-old European-American children [3]. A 2018 systematic review showed that children of immigrant and refugee backgrounds living in Australia were more likely to be diagnosed with neurodevelopmental disabilities, particularly autism with co-occurring ID [18]. This was also found in a population-based Australian study that investigated the prevalence of developmental disabilities, finding that the impact of maternal migration acted in two ways—it increased the likelihood factor of diagnosis with autism with ID for children of non-Australian-born women from low- and middle-income countries, but was also as a protective factor for children of non-Australian-born mothers for diagnosis of developmental disabilities overall [19]. This was further evident in a 2022 systematic review on migration and autism prevalence, which found maternal migration increased the likelihood of an autism diagnosis, with migrant autistic children more likely to also be diagnosed with co-occurring ID [20].

Several studies have investigated biological and environmental markers of autism with stronger evidence for the role of maternal factors [21,22]. For children of migrant mothers, it has been hypothesized that the epigenetics of pregnancy, maternal stress, the possible role of vitamin D deficiency, and maternal migration could result in poor intrauterine growth, pregnancy complications, and developmental differences [23,24,25,26]. These studies have also suggested that maternal and perinatal factors play a critical role in the increased likelihood of autism, with differences observed according to maternal country of birth and ethnicity [25,27]. In addition, the conditions and nature of migration have also been identified as associated with diagnosis of developmental disabilities such as autism and particularly autism with ID [26,28]. Factors such as cultural differences in child development, childrearing practices, the stigma of having a disabled child, lack of access to translators, clinician bias, and misinformation can further complicate these dynamics and also lead to misdiagnosis [23]. Post-migration and resettlement factors, such as educational attainment, unemployment, housing insecurity, and access to affordable health services, add to these complicated dynamics [29].

### 1.1. Socioeconomic Status

The social determinants of health—the circumstances that people are born into and live, grow, work, and play—exponentially predict an individual’s health quality and outcomes [29]. Globally, social systems and structures further deepen these differences and unequally distribute the social determinants of health into social determinants of health inequities [30]. Health inequities describe the differences in health outcomes and risk factors between inter- and intra-groups. The direct relationship between Social Economic Status (SES) and autism prevalence is inconclusive; some studies have reported association between higher prevalence of autism with higher SES in US, whilst other studies observed a higher prevalence of autism in lower SES regions in some parts of Europe [4,10,31]. Research has focused on SES, autism prevalence, and health disparities in France and the US, demonstrating the complexities of these relationships [10,11]. For example, Delobel-Ayoub and colleagues’ study in France found a significantly higher prevalence of autism with ID in the lowest SES areas marked by high unemployment rates, low educational attainment, and immigrant and single-parent household status [10]. In contrast, autism without ID had significantly higher prevalence in areas with the highest immigrant status. This study suggested significant health disparities were contributing factors to its findings, as despite France’s universal free health coverage, substantial socioeconomic and health inequalities in child health were evident [10]. Durkin and colleagues also suggested significant healthcare disparities in access to diagnostic and support services for autistic children in explaining the relationship between increasing autism prevalence and higher SES, particularly in children with a pre-existing autism diagnosis [11].

In Australia, one principal avenue to observe SES and health inequities is the Australian Bureau of Statistics (ABS) definition of socioeconomic status (SES) as household income, educational attainment, and employment, as well as its Socio-Economic Indexes for Areas (SEIFA) measure [32]. SEIFA is a set of indexes created from the Australian Census summarising the diverse population, family, and household characteristics related to socioeconomic advantages and disadvantages collected in the Census of Population and Housing and provides a ranking of all areas in Australia. The Index of Relative Socio-Economic Advantage and Disadvantage (IRSAD), one set of indexes from SEIFA, is a general measure of relative SES. 

### 1.2. Australian Healthcare System

The Australian healthcare system operates on a universal healthcare model. Due to underfunding by federal and state-level governments, waitlists for public services are extensive. While private services with reduced waiting times are available, these are very costly for families, often resulting in a delayed age at diagnosis for autism [33]. However, research engagement can provide opportunities for timely diagnostic assessments, address parental/caregiver concerns regarding their child’s development, and provide guidance and navigation for access to early supports. Thus, families who engage with research may avoid long public health system waitlists, costly private clinic assessments, and delayed diagnosis. The mean age at diagnosis in Australia is around 4.1 years in children under 7 years [34], which is consistent with a recent systematic review that attempted to ascertain the global average age at autism diagnosis of 3.6 years [35]. Current evidence and best practice support accurate and effective diagnosis age at two years [34,35], thus providing a critical opportunity to take advantage of early therapy and support, which can positively change the trajectory of child outcomes [36]. In Victoria, Australia, maternal and child health (MCH) nurses monitor children from birth to preschool age across 12 scheduled ‘key ages and stages’ well-child visits, unless there are concerns for the child’s development, which often warrant extra visits [37]. As a result, the Victorian MCH service has the highest well-child visit attendance rates across Australia, with participation rates of 83.1% at 12 months, 74.2% at 18 months, 70.6% at 24 months, and 64.2% at 42 months [37]. These free, universally available MCH visits are vital for monitoring child health and development, including for autism. In addition, MCH nurses have extensive knowledge and formal training on developmental surveillance and milestones. 

The Social Attention and Communication Surveillance-Revised and Preschool (SACS-R+PR) study was a large community-based study conducted from 2013 to 2018 in eight local government areas (LGAs) in Victoria, Australia, whereby 126 MCH nurses were trained in the early identification of autism and monitored 13,511 infants and toddlers from the general population at their 12-, 18-, 24-, and 42-month well-child checks [8]. Children with a “high likelihood” for an autism diagnosis were referred by the MCH nurses to attend free standardised diagnostic assessments by the La Trobe University research team. The current study uses retrospective data from the SACS-R+PR study to address gaps in the literature exploring potential differences between diverse groups engaging with research opportunities. For the purpose of this study, research engagement is defined as family acceptance of referral from MCH nurses and undertaking the diagnostic assessment at the university research centre. This retrospective study aimed to explore research engagement of families by investigating the associations between (1) family ethnic background and research engagement, and by (2) comparing autism prevalence across groups according to family ethnic background against their represented population at each LGA.

## 2. Materials and Methods

This current study is a retrospective study of the original SACS-R+PR study, and for further methodological detail, refer to the initial study [8].

### 2.1. Study Design

This study examined associations between research engagement, an autism diagnosis, and several socioeconomic status indicators from our study data (alongside ABS IRSAD score), such as maternal educational attainment, employment, household income, language(s) spoken at home, and socioeconomic score (SEIFA—IRSAD) per LGA. This study utilized existing data collected from the SACS-R+PR cohort by LGAs and data published in the Australian 2016 Census [38] on these LGAs.

### 2.2. Local Government Area Demographics

**Figure 1:**
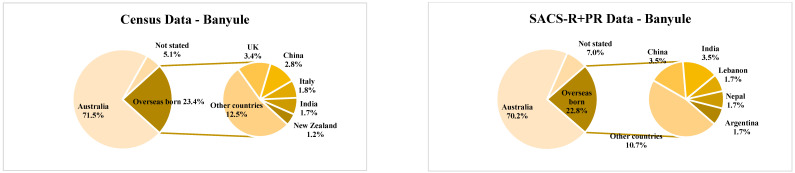

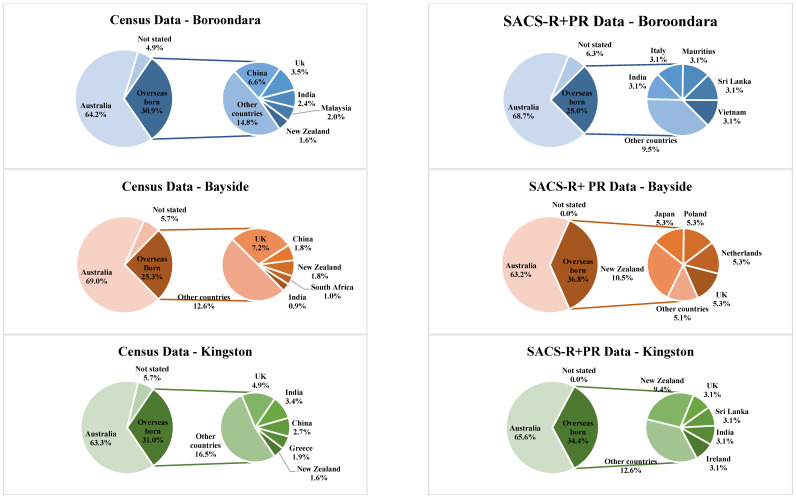

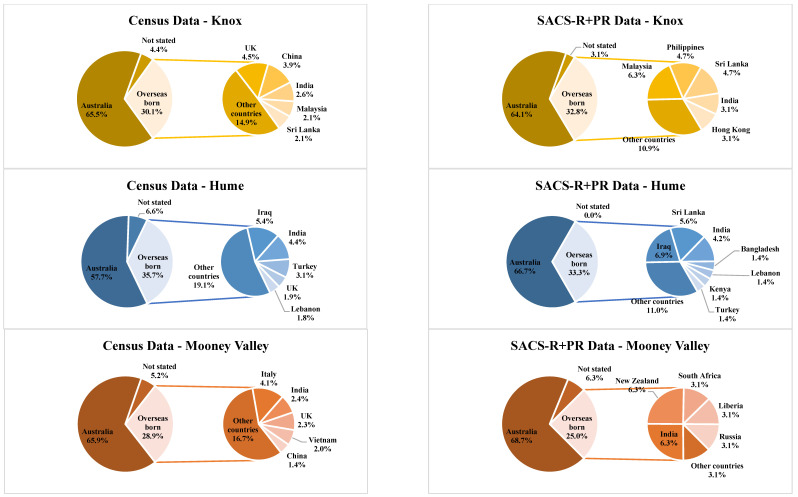

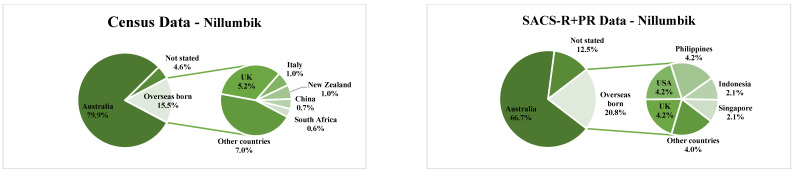
Population country of birth (Australian 2016 census) and maternal country of birth (Social Attention and Communication Surveillance-Revised + Preschool data) by local government area. Note: Census; Australian 2016 census data. SACS-R+PR; Social Attention and Communication Surveillance-Revised + Preschool data.

**Figure 2:**
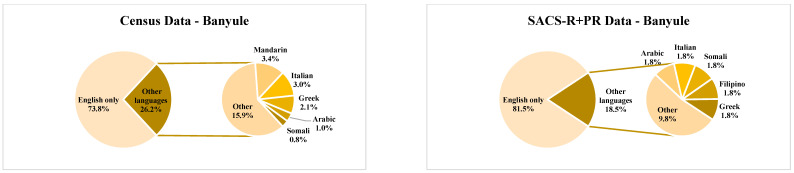

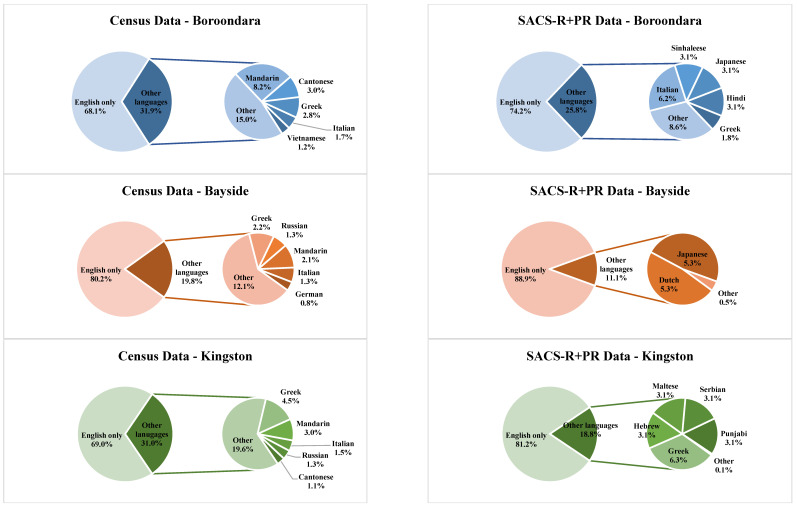

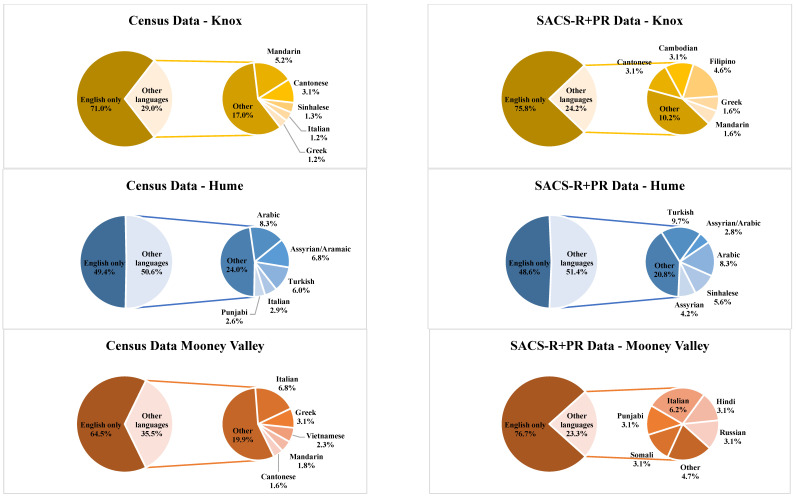

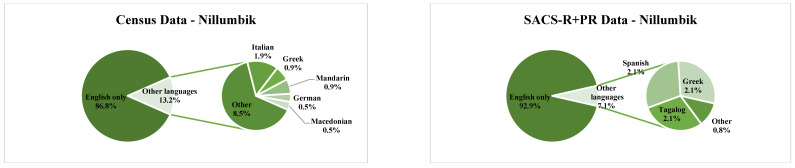
Languages spoken at home by local government area according to the Australian 2016 census and the Social Attention and Communication Surveillance-Revised + Preschool data. Note: Census; Australian 2016 census data. SACS-R+PR; Social Attention and Communication Surveillance-Revised + Preschool data.

The eight LGAs included in this study—Banyule, Bayside, Boroondara, Hume, Kingston, Knox, Moonee Valley, and Nillumbik—are located across the north, northwest, south, and east of metropolitan Melbourne and were recruited to the SACS-R+PR study due to their high attendance rates at the 42-month MCH well-child visits at the time of study commencement as well as proximity to the university to enable easier access for developmental assessments for families [8]. These communities’ geographic and population demographics according to the Australian 2016 census [38] are listed in Table 1. Population sizes ranged from the lowest in Nillumbik (n = 61,273) to the highest in Hume (n = 197,671; Table 1). The percentage of individuals born in Australia in each LGA ranged from 57.7% in Hume to 79.9% in Nillumbik (Figure 1). In addition, the percentage of overseas-born individuals residing in each LGA ranged from the lowest in Nillumbik (15.5%) to the highest in Hume (35.7%), with the most common countries of birth across the LGA’s being Iraq, the UK, India, Turkey, and China (Figure 1). Much of the population spoke English only; however, 44.8% of Hume residents and 29.8% of Moonee Valley residents spoke a non-English language (either exclusively or in addition to English), and overall, the most commonly spoken languages were Arabic, Assyrian, Turkish, Italian, Greek, Punjabi, and Vietnamese (Figure 2; [38]). The percentage of low-income households (household income of less than AUD 650 per week) was higher in Kingston (18.7%), Moonee Valley (18.5%), and Hume (18.4%), whilst high-income households (household income of AUD 3000 and above per week) were more common in Bayside (35.8%) and Boroondara (33.8%; Table 1). Most SEIFA scores in the LGAs had an IRSAD score within the standardized mean score (standardized against a mean of 1000 with a standard deviation of 100), except for Boroondara and Bayside, who were above the SEIFA standardized mean score, scoring 1128 and 1125, respectively, while Hume was positioned at the lower end of the mean with a score of 947.

### 2.3. Study Recruitment

**Figure 3:**
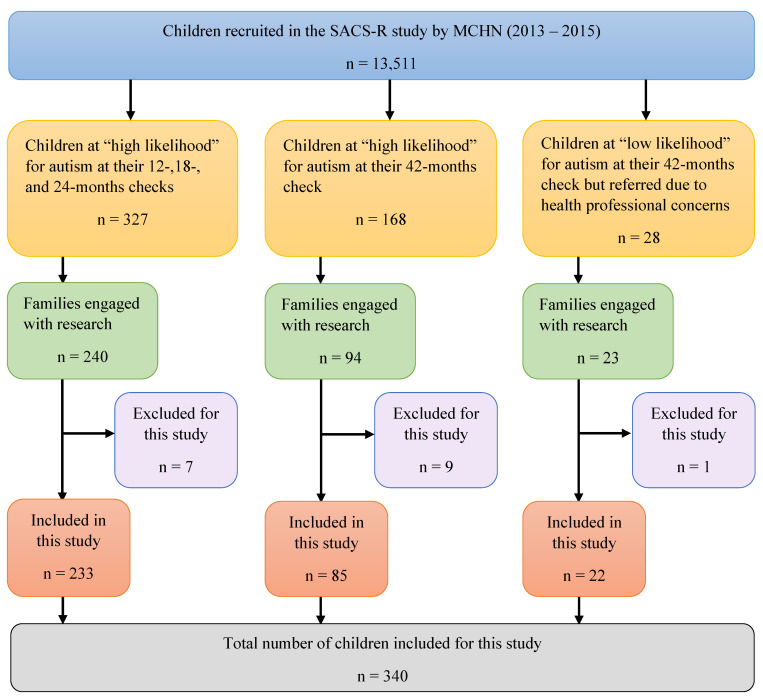
Flowchart of the study inclusion criteria.

The study design controlled for many impeding barriers. Once a child was identified with a high likelihood for autism, families were referred to the university research centre for free diagnostic assessments. Relevant accommodations were made to enable full participation based on families’ circumstances, such as translation of study documentation, interpreters for families during the assessments, and taxi vouchers for those without adequate transport. During the SACS-R+PR study, MCH nurses recruited 13,511 children aged 11 to 30 months from the above-mentioned LGAs, using opt-out recruitment. The greatest proportions of children were residing in Knox (n = 2617; 19.37%), Boroondara (n = 2510; 18.58%), and Hume (n = 1925; 14.25%; Table 2). The MCH nurses monitored the children for early signs of autism using SACS-R at their 12- to 24-month health checks and followed up with the cohort at their 42-month health check using the SACS-PR tool. As a result, 327 children were identified as having a high likelihood for autism between 11 and 30 months of age, and 168 children were identified between 31 and 54 months of age, with all children referred to the study team for assessment. An additional 28 children aged 31 months and over were referred to the study team at 42 months due to parental/caregiver and/or MCH nurse concerns (see Figure 3). In all, 523 children were referred to the study team for assessment as part of this study. Of these, families of 357 children accepted the referral and attended at least one diagnostic assessment; tools administered included the Autism Diagnostic Observation Schedule-Section Edition (ADOS-2; modules 1–3) or the ADOS-Toddler (ADOS-T) [39,40], Mullen Scales of Early Learning [41], and a brief developmental interview or the Autism Diagnostic Interview-Revised (ADI-R) [42], dependent on the child’s age. The diagnostic outcome of the 357 children was autism (n = 268; 75.07%), and developmental and/or language delay (n = 89; 24.93%) [8].

Participating families completed a comprehensive demographic questionnaire at the diagnostic assessment. The inclusion criteria for the current analysis were as follows: children from the SACS-R+PR study who attended at least one university diagnostic assessment; the child’s caregiver had completed at least 50% of items in the demographic questionnaire on maternal ethnicity and maternal country of birth, along with other variables such as highest level of maternal education attained, language(s) spoken at home, and annual family income; and the caregiver had given informed consent for the use of their data in future research. Participants with missing data were excluded from analysis. A total of 340 SACS-R+PR families fit our inclusion criteria and were included in this analysis, ranging from 19 (5.6%) in Bayside to 72 (21.2%) in Hume. 

### 2.4. Maternal and Child Groups

Maternal ethnicity was used to formulate “ethnicity” in the current study and grouped according to the ABS’s Classification of Cultural and Ethnic Groups (ASCCEG) [43]. Ethnicity or ethnic background can have many meanings; however, ASCCEG defines ethnicity as the shared identity or similarities of a group of people based on one or more of the following distinguishing characteristics: shared history, cultural tradition, geographic origin, common language, common literature, common religion, being of a minority ethnicity, and being racially conspicuous [43]. 

**Figure 4:**
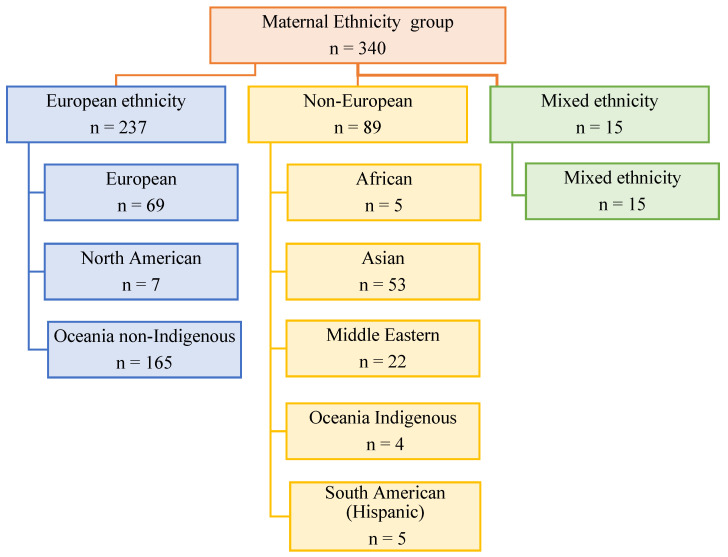
Child ethnicity grouping according to the combination of maternal ethnicities.

Although these groups help us to further group potentially ‘like’ groups and populations, the authors note that no assumptions were made of these groups as being homogenous, but as members that do share some similarities, and thus, these categories, along with the Australian 2016 census data (see Table 1, and Figure 1 and Figure 2) were used in an attempt to illustrate potential differences of opinions and outcomes amongst caregivers from each of these groups. The percentage of Australian-born mothers in each LGA ranged from the lowest in Bayside (63.2%; n = 12) to the highest in Banyule (70.2%; n = 40). In comparison, the lowest percentage of overseas-born mothers resided in Nillumbik (20.8%) compared to the highest number of overseas-born mothers residing in Bayside (36.8%), most commonly from Iraq, the UK, India, Turkey, and China (Figure 1). The percentage of multilingual SACS-R+PR mothers was highest in Hume (51.4%), followed by Boroondara (25.8%). The most common non-English languages spoken at home by SACS-R+PR families overall were Turkish, Arabic, Sinhalese, Italian, Assyrian, and Japanese (Figure 2). In this study, maternal ethnicity was combined with maternal country of birth and sub-classified into the following seven ethnicities: Australia, New Zealand, and the surrounding Oceania region (Oceanian non-Indigenous (self-identified as Caucasian; n = 165), Oceanian Indigenous (self-identified as Aboriginal/Torres strait Islander or Māori; n = 4); the continent of Europe (European; n = 65); the continent of Africa (African; n = 5); North America (self-identified as Caucasian; n = 2) and South America (Hispanic; n = 7); Central Southern, South East, and North East Asia (Asian; n = 53); the Southern and Eastern shores of the Mediterranean Sea (Middle Eastern; n = 22); and a group inclusive of any combination of these mentioned ethnicities (Mixed ethnicity; n = 15). These seven ethnicities were further grouped as ‘European Maternal Ethnicity’ (EME; European and non-Indigenous Oceanian only), ‘non-European Maternal Ethnicity’ (N-EME; African, Asian, and Middle Eastern only) and ‘Mixed Maternal Ethnicity’ (MME; both European and non-European maternal ethnicity) (see Figure 4). 

### 2.5. Analysis 

For this study, we compared the SACS-R+PR demographic data against the data obtained from the Australian 2016 census to ascertain whether the convenience sample of participants in this research were representative of their corresponding LGA. Descriptive statistics were used to characterize LGA, child, and maternal demographics, and the outcome of a diagnostic assessment and were completed using Stata 6.1 [44]. The maternal demographic variables included ethnicity, educational background, and annual family income (in Australian dollars, AUD), as described in Table 3. Child variables consisted of child gender, country of birth, and LGA of residence (see Table 3).

Child clinical outcomes of overall diagnosis consisted of autism or developmental and/or language delay (DD/LD)—no child identified by the SACS-R+PR was typically developing. Pearson pairwise correlation analyses were conducted to examine relationships between the measures for each group separately. In addition, multinomial logistic regression analysis examining the crude and adjusted relative risk ratios (cRRR, aRRR) of an autism diagnosis compared to DD/LD was conducted for children of N-EME and MME compared to EME, and RRR with 95% confidence intervals were calculated.

## 3. Results

### 3.1. Local Government Area Statistics

**Figure 5:**
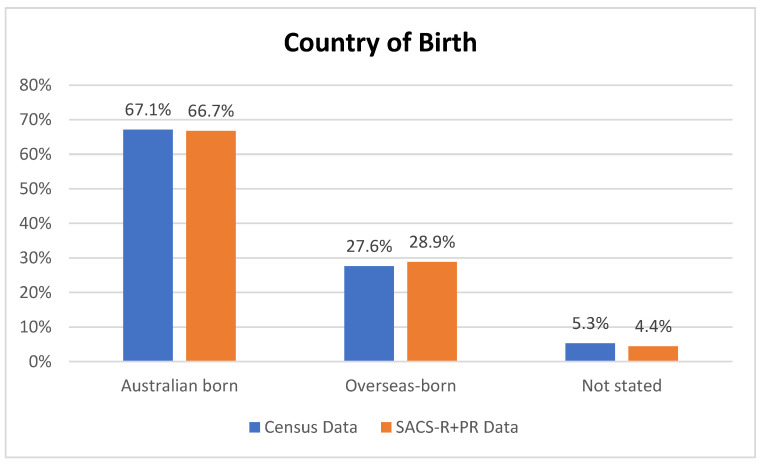
Country of birth for participating local government areas (from Australian 2016 census data) compared to SACS-R+PR data.

**Figure 6:**
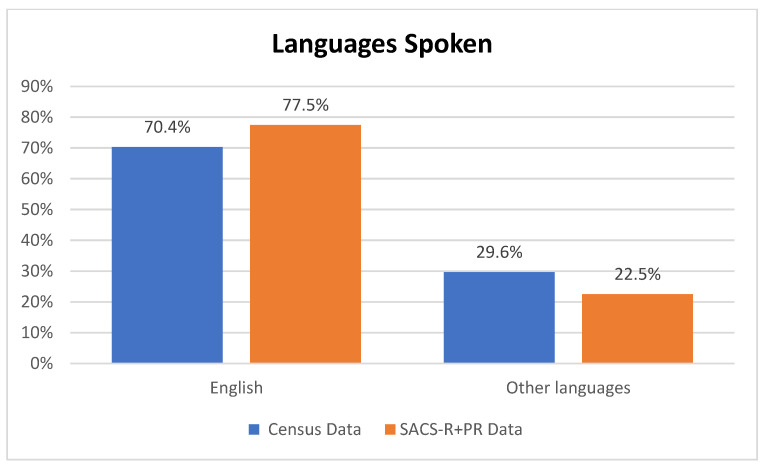
Overall languages spoken at home for participating local government areas (from Australian 2016 census data) compared to SACS-R+PR data.

The average percentage from the census for the overall combined population from the eight LGAs was as follows: 67.12% born in Australia, 27.6% born overseas, and 5.28% not stated. A breakdown of the SACS-R+PR population is as follows: 66.74% born in Australia, 28.86% born overseas, and 4.40 not stated (Figure 5). Overall, there were no differences between the proportion of the Australian-born population and overseas-born between the Australian 2016 census data and the families who participated in the SACS-R+PR research (Z-ratio = −0.505, *p* = 0.3068). Complete data sets per LGAs, including the percentages of the top five countries for overseas-born residents and corresponding participants from the SACS-R+PR compared to respective LGA populations, are found in Figure 1. In comparing monolingual and multilingual languages spoken, the census lists multilingual languages ranking them from most spoken (the top 5) to not stated: 70.35% English only, 13.15% multilingual, and 16.50% not stated. A breakdown of the SACS-R+PR population in terms of languages is as follows: 77.48% English only, 15.73% multilingual, and 6.80% not stated. The proportion of families from the study speaking multiple languages to English-only speakers was slightly higher than the represented LGA communities who were more likely to speak English only (Z-ratio = 1.33, *p* = 0.09; Figure 6). Similarly, complete data sets per LGA, including the proportion of residents speaking of the top five languages and corresponding participants from the SACS-R+PR compared to the respective LGA population, are found in Figure 2. 

### 3.2. SACS-R+PR Recruitment Demographics

**Figure 7:**
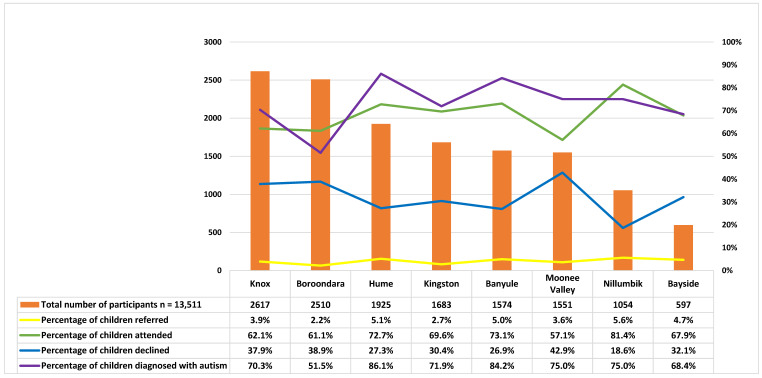
Percentages of children referred, attended, declined, and diagnosed per local government areas.

The MCH nurses recruited children into the study across three age groups: 12 months age bracket (n = 6458, 47.8%), 18 months age bracket (n = 3890, 28.8%), and 24 months age bracket (n = 3163, 23.4%) (Table 2). This cohort was followed up at 6-month intervals up to 30 months and again at 42 months (Figure 3). The majority of participating children (n = 10,701, 79.2%) attended multiple MCH assessments, with only 20.8% (n = 2810) of children attending only one MCH assessment (Table 2). The nurses completed 31,708 SACS-R+PR assessments throughout the study: 6458 at 12 months, 7830 at 18 months, 9001 at 24 months, and 8419 at 42 months (Table 2). The percentage of children identified at high likelihood for autism in each age bracket by LGA are listed respectively from highest to lowest: Nillumbik (n = 59, 5.6%) to Boroondara (n = 54, 2.2%) (Table 2). Nillumbik also had the highest research attendance rate (n = 48, 81.3%), whilst Hume had the highest percentage of autism diagnosis (Table 2 and Figure 7). The four LGAs with the highest proportion of attending families corresponding to the number of referrals were as follows: Nillumbik (n = 48, 81.3%), Banyule (n = 57, 73.0%), Hume (n = 72, 72.7%), and Kingston (n = 32, 69.5%).

### 3.3. Maternal and Child Descriptive Statistics

Maternal ethnicity information was available for 95.2% (n = 340) of participants, and of these, 69.4% (n = 236) were of EME, 4.4% (n = 15) of MME, and 26.2% (n = 89) of N-EME (Table 3). Our sample had more males compared to females (n = 279, 78.1%, vs. n = 78, 22.9%). There was representation from all ethnic groups across the LGAs except for Banyule, with no children from the MME group (see Figure 6). The LGAs with the greatest proportions of children of N-EME were Hume, Knox, and Banyule; children of MME were Hume, Boroondara, and Knox; and children of EME were Knox, Banyule, and Nillumbik (*p* < 0.001, Table 3). English was the dominant language in children of EME (89.0%, n = 210) and MME (86.7%, n = 13) in comparison to N-EME (31.5%, n = 28). However, multilingualism was significantly higher in children of N-EME (57.3%, n = 51) compared to children of MME (13.3%, n = 2) and of EME (9.3%, n = 22) (*p* < 0.001, Table 3). Differences were observed for children according to ethnicity in maternal employment status. Mothers of children of N-EME had a higher rate of current employment (51.7%, n = 46) than children of EME (45.7%, n = 108) or MME (40.0%, n = 6) (*p* = 0.025). Differences were also observed in maternal education, with mothers of N-EME children most likely to have university degrees at (72.6%, n = 66) compared to MME (66.7, n = 10%) and EME (52.5%, n = 124) mothers (*p* = 0.018). Higher annual family incomes were observed for MME (n = 4, 26.7%) and EME (15.3%, n = 36) children compared to N-EME (9.0%, n = 8) (*p* < 0.001, Table 3).

### 3.4. Child Clinical Outcomes

Of the 340 participants included in this study, 75.1% (n = 268) children received a diagnosis of autism (N-EME: n = 73, 82.0%; EME: n = 177, 75.0%; MME: n = 7, 46.7.%) (Table 3). Whilst 24.4% (n = 83) of children were diagnosed with DD/LD (MME: n = 8, 53.3%; EME: n = 60, 24.9%; N-EME: n = 15, 17.9%) (Table 3). Significant correlations between child, maternal demographics, and child outcomes were observed. The most notable differences in the patterns of associations among the three groups are shown in Supplementary Tables S1–S4. Interestingly, four significant associations were observed both for children of N-EME and EME, compared to three for children of MME. Multinomial logistic regression analysis of the three maternal ethnicity groups observed the RRR of a diagnosis of autism compared to DD/LD diagnosis. Children of MME had a significantly lower likelihood of receiving an autism diagnosis than a DD/LD diagnosis compared to children of EME (cRRR 0.29, 95% CI 0.10, 0.83, *p* = 0.002, and aRRR 0.26, 95% CI 0.09, 0.78, *p* = 0.017). However, whilst not statistically significant, children of N-EME had a 1.5-fold likelihood of an autism diagnosis rather than a DD/LD diagnosis compared to children of EME (cRRR 1.52, 95% CI 0.81- 2.86, *p* = 0.189) and a 1.3-fold likelihood upon adjustments for maternal education, employment, annual family income, and languages spoken at home (aRRR 1.33, 95% CI 0.60–2.92, *p* = 0.480) (Table 4).

## 4. Discussion

There is limited research examining potential differences among diverse groups’ engagement with research opportunities and even more so in the early identification and diagnosis of autism. This study examined the role of ethnicity and family background in research engagement in proportion to respective local communities and explored if ethnicity and SES contribute to the diagnosis rate of autism. SACS-R+PR had an excellent overall representation from each LGA when compared to community-level data. For example, in observing migrant mothers in our sample, there was no difference in attendance between the proportion of Australian-born and overseas-born populations between the LGAs and the families engaged in the research. However, there was a slightly higher proportion of families in the study who spoke multiple languages including English (rather than English only), than the represented LGA communities speaking multiple languages in proportion to English. 

Furthermore, our study consisted of a strong cohort of 73.4% of families with a child identified at high likelihood for autism at their MCH visit that engaged with research services. Diagnosis per gender was consistent with the literature with more males than females at 3.4 to 1. Our findings demonstrated demographic disparities and showed a relationship between family background and research engagement. This study also demonstrated that research engagement was driven by high maternal education, SES, annual family income, and current maternal employment. Whilst autism prevalence in the current study was greater in children of N-EME compared to EME, the RRR was insignificant.

### 4.1. Prevalence

The current study found an increased proportion of children of N-EME with referral for high-likelihood of autism, diagnosed with autism, more likely to speak two or more languages at home, and were more likely to reside in areas such as Hume, marked by high numbers of cultural and linguistical diversity as well as pockets of significant disadvantage. In the SACS-R+PR study, Hume had the second highest MCH attendance and the highest percentage of an overall autism diagnosis in our study. The association of increased proportions of children with an overall diagnosis of autism from all age brackets in our current study demonstrates the importance of professional competence, support and alignment with research services, the families’ awareness of the early signs of autism, and willingness to be involved in developmental screening once presented with the opportunity [45]. Evidently, the success of the SACS-R+PR study the effective training of MCH nurses on the early signs of autism [8], and the free diagnostic assessments offered as part of this study enabled N-EME children in areas of low SES to be successfully identified and diagnosed early, thus bridging existing disparities for this cohort and going against the findings that autism is particularly more prevalent in areas of high SES [46].

### 4.2. Maternal Employment and Language

In our study, children of N-EME had higher rates of current maternal employment than expected and were significantly more likely to speak two or more languages at home including English, compared to English only, than children of MME and EME. Furthermore, children of N-EME had a significantly higher prevalence of autism diagnosis within the study. A significant majority resided in areas of low SES, which was consistent with previous research from France, which found the highest prevalence of autism in areas of low SES and the highest percentages of immigrants [10] but contradicts a 2017 US study that found low SES areas had lower autism diagnosis prevalence. The Durkin et al. 2017 study echoed healthcare system inequities faced by families in the US, a typical scenario of children with low SES not having access to diagnostic services [47]. However, our study and the French study were in line with similarities in the two healthcare systems and thus demonstrating how our study design essentially leveraged off the universal approach in healthcare with the addition of targeted developmental surveillance for autism, thus enabling timely and accurate diagnosis for children residing in lower SES areas and of minority backgrounds [48]. 

### 4.3. Maternal Education and Annual Family Income

Correlation and multinomial regression analysis were conducted to determine associations and drivers of research engagement and child outcomes of autism diagnosis. Maternal employment alongside maternal education, SEIFA, and annual family income significantly drove research engagement. This finding for EME children being from areas of high SES, education attainment and medium to high annual family incomes, are consistent previous research on high SES and autism diagnosis [11,47,49]. These findings of greater SES status for children and their families of European ethnicity, are unsurprising and demonstrate social gradient. More resourced, advantaged, and educated families are more likely to navigate the healthcare system with greater ease and more likely to engage in research studies to aid in gaining a diagnosis for their child following concerns either by parents or professionals. Whilst the literature on SES and autism has often been mixed and inconclusive [10,11,31,46], with positive and negative associations between autism prevalence and SES, our study determined that SES (defined as maternal employment, education, and family income) was a driving force for research engagement in such families.

For MME and N-EME children, research engagement was driven significantly by, current maternal employment, high maternal education, and multiple languages spoken at home. However, for N-EME children, as families SEIFA increased there was a negative association of a decrease of multilingualism at home. Also in our study, children of an ethnic minority or mixed ethnicity backgrounds were more likely to engage with research as a result of increased education attainment, and medium annual family income, and N-EME children had increased relative risk of an autism diagnosis which was statistically not significant but was consistent with previous research [11,49,50].

### 4.4. Ethnicity

This study’s findings showed a relationship between N-EME children and the prevalence of autism, which is consistent with current research [10,14,18,51]. Correlation and multinomial logistic regression analysis were conducted to determine drivers of research engagement and child outcomes of autism diagnosis. Whilst the autism prevalence was different between our ethnicity groups, the relative” high likelihood” of an autism diagnosis compared to a DD/LD diagnosis that was 1.5 (crude ratio) and 1.3 times higher for children of N-EME compared to EME was statistically non-significant. This non-significance is protectively suggestive of the streamline nature of the SACS-R+PR cohort as well as how highly educated and resourced families valuing and accessing research services in obtaining a timely diagnosis. Data from the ADDM Networks 11 sites recently demonstrated that this difference in overall prevalence between children according to their race and ethnicity has reduced, indicating the importance of awareness and access to early diagnosis, with only Hispanic children at specific sites less likely to be identified as having autism than European-American or African American children [19]. However, the proportion of African American autistic children with ID was disproportionately higher. The literature on autism prevalence has demonstrated variations globally, within countries, communities, and groups [52,53]. Our current study found statistically significant differences in prevalence among children according to ethnicity and social determinants of health. Other studies have also identified such differences [17,18,51]. The authors call for further research, as the nature of the research study with a streamlined diagnostic pathway and the universal availability of MCH services for developmental screening, including monitoring children for the early signs of autism across these eight communities, was able to reduce the prevalence disparities in early identification and diagnosis of autism in the study. Conversely, progress has been made over time in the early identification and diagnosis of autism [54], with extensive parental education, awareness, training of professionals, and early diagnosis; however, globally, there remain significant differences in the early identification of autism by race and ethnic groups.

Nevertheless, this study in the State of Victoria, Australia, controlled for many impeding factors, obstacles, and waiting times [55] to early identification and diagnosis, such as training of MCH nurses on the early signs, use of an evidence-based and highly sensitive developmental surveillance tool (SACS-R+PR), and direct referral pathways that enabled the immediate attention and assessment of a child’s developmental concerns free of cost. 

### 4.5. Limitations and Future Research Directions

This study had shown some significant strengths: namely, a strong cohort of 73% of participant referrals engaging in research when identified as having a "high likelihood" of autism. The cohort also formed a convenient sample demonstrating a representative population of the corresponding LGAs, thus ensuring that this targeted approach reached typically underrepresented groups in LGAs such as Hume. Another strength was that the geographical locations of the LGAs made it possible for participants to engage in research. However, whilst this study had significant strengths in the early autism identification and diagnosis of children in a community-based setting, a few limitations should be noted. Firstly, data collection from the ABS pertaining to education and socioeconomic status differed slightly from the question items collected in the SACS-R+PR demographic questionnaires, making it harder to compare participants to their corresponding communities. Secondly, accurately demonstrating disadvantage in Australia, using SEIFA comparatively is somewhat complex and challenging, as IRSAD measures variables broadly reflecting disadvantages and advantages at the geographical area level and not at the individual level our study data consisted of [32]. Due to the complexities of IRSAD, SEIFA indexes, and social housing in Australia, disadvantaged areas can be embedded within advantaged areas, such that there are often pockets of disadvantage. This means that individuals of relative advantage can live in areas of disadvantage, and thus, profiles of disadvantage within Australia often mean that disadvantage is deeply hidden within advantaged areas [38]. For example, there are pockets of poverty, unemployment, low education, and social housing settlement within significantly advantaged areas. Despite this complexity, it can explain our cohort’s high MCH attendance and research engagement. Thirdly, as mentioned above, the nature of the SACS-R+PR study was that MCH attendance allowed for fast access to free diagnostic assessments. [56,57]. However, some families did not attend the free available MCH services and some families in high SES areas who declined research engagement, bypassed the MCHN referral to the study and sought services from private multi-disciplinary teams and paediatricians. Fourthly, we did not have data for 166 children deemed "high likelihood" due to a decline of referrals and some missing data from the questionnaires impacted data collection and our analysis. Finally, whilst significant provisions and efforts of engaging predominately disadvantaged families in local MCH services were made and study adjustments were put in place, we are unable to account for or have information about factors that continue to still act as barriers to early diagnosis; the impacts of culture differences, assumptions of “normal” child development, stigma for disability, and having a disabled child significantly prevents families from seeking and accessing relevant services. This SACS-R+PR study had significant strengths in successfully enabling the identification and diagnosis of all children, and it is a model for early identification and diagnosis with its universal and targeted approaches. Future directions include but are not limited to mapping out data across a wider community sample and educating the community on the mutual benefits of research engagement: families benefit from free assessments and scientific advances in knowledge.

## 5. Conclusions

Children of ethnic minority backgrounds were more likely to be identified at “high likelihood” for autism and be diagnosed with autism. The SACS-R+PR study showed how the targeted approach of community-based developmental surveillance for autism, coupled with trained health professionals and streamlined referral pathways, can ensure the early identification and diagnosis of autism for all children regardless of family background. Streamlined universal evidence-based early autism screening and timely referral lead to equitable access to diagnosis and can bridge disparities in the communities. The SACS-R+PR study models improved and optimal care in child developmental services. It effectively showed how universal and targeted approaches to autism could bridge the gaps in identifying and diagnosing autism for minority communities.

## Figures and Tables

**Table 1:** Geographic and population demographics of the local government areas included according to the census data and SACS-R+PR maternal ethnicity.

			Banyule	Bayside	Boroondara	Kingston	Knox	Hume	Moonee Valley	Nillumbik
Location			North-Eastern	Southern	Eastern	South-Eastern	Eastern	North-Western	North-Western	Northern
Population	Census		121,865	97,087	167,231	151,389	154,110	197,376	116,671	61,273
			M: 48.6%	M: 47.6%	M: 47.9%	M: 48.6%	M: 48.9%	M: 49.8%	M: 48.3%	M: 49.4%
			F: 51.4%	F: 52.4%	F: 52.1%.	F: 51.4%	F: 51.1%	F:50.2%	F: 51.7%	F: 50.6%
	Study		57	19	32	32	64	72	32	48
			M: 86.0%	M: 79.0%	M: 81.3%	M: 87.5%	M: 76.6%	M: 66.7%	M: 84.4%	M: 77.1%
			F: 14.0%	F: 21.0%	F: 18.7%	F: 12.5%	F: 23.4%	F: 33.3%	F: 15.6%	F: 22.9%
Aboriginal and Torres Strait Islander peoples	Census		0.6%	0.2%	0.2%	0.4%	0.5%	0.7%	0.4%	0.4%
	Study		0.0%	0.0%	0.0%	0.0%	3.2%	0.0%	6.3%	0.0%
Household Income in Australian dollars	Census	Low	17.0%	15.4%	15.4%	18.7%	16.2%	18.4%	18.5%	11.1%
		High	20.1%	35.8%	33.8%	17.4%	15.5%	11.2%	22.1%	28.4%
	Study	Low	10.5%	10.5%	0.0%	3.1%	7.8%	15.3%	9.4%	8.3%
		High	10.5%	31.2%	37.5%	15.6%	4.7%	5.6%	6.3%	20.8%
IRSAD	Census		1055	1125	1128	1042	1032	947	1046	1093

Notes: Source: Australian 2016 census (community-level profile). Index of Relative Socioeconomic Advantage and Disadvantage (IRSAD), the standardized mean score (standardized against a mean of 1000 with a standard deviation of 100); Household Income in Australian dollars (low; <$650 per week, high; >$3000 per week).

**Table 2:** Participant recruitment age brackets, attendance at the maternal and child health visits, and identification of high likelihood for autism by local government area.

	Banyule		Bayside		Boroondara		Hume		Kingston		Knox		MooneeValley		Nillumbik		Total	
	n	%	n	%	n	%	n	%	n	%	n	%	n	%	n	%	n	%
Number of children monitored at each age bracket																		
12 months	814	51.72	212	35.51	1195	47.61	902	46.86	746	44.33	1253	47.88	812	52.35	524	49.72	6458	47.80
18 months	439	27.89	213	35.68	730	29.08	576	29.92	489	29.06	742	28.35	417	26.89	284	26.94	3890	28.79
24 months	321	20.39	172	28.81	585	23.31	447	23.22	448	26.62	622	23.77	322	20.76	246	23.34	3163	23.41
Total	1574	100.00	597	100.00	2510	100.00	1925	100.00	1683	100.00	2617	100.00	1551	100.00	1054	100.00	13,511	100.00
Number of children with only a single engagement with the MCH nurse at each age bracket																		
12 months	167	47.99	48	40.00	117	32.68	291	42.42	309	52.46	122	31.12	86	39.81	42	42.00	1182	42.06
18 months	94	27.01	29	24.17	95	26.54	201	29.30	138	23.43	125	31.89	53	24.54	23	23.00	759	27.01
24 months	87	25.00	43	35.83	146	40.78	194	28.28	142	24.11	145	36.99	77	35.65	35	35.00	869	30.93
Total	348	100.00	120	100.00	358	100.00	686	100.00	589	100.00	392	100.00	216	100.00	100	100.00	2810	100.00
Number of attendances at the MCH checks overall																		
Single	348	22.11	120	20.10	358	14.26	686	35.64	589	35.00	392	14.98	216	13.93	101	9.58	2810	20.80
Multiple	1226	77.89	477	79.90	2152	85.74	1239	64.36	1094	65.00	2225	85.02	1335	86.07	953	90.42	10,701	79.20
Total	1574	100.00	597	100.00	2510	100.00	1925	100.00	1683	100.00	2617	100.00	1551	100.00	1054	100.00	13,511	100.00
Number of assessments by the MCH nurse at each age bracket																		
12 months	814	21.87	212	15.33	1195	18.63	902	24.04	746	23.38	1253	19.26	812	20.39	524	19.01	6458	20.37
18 months	939	25.23	321	23.21	1617	25.20	996	26.55	715	22.41	1578	24.26	985	24.74	679	24.63	7830	24.69
24 months	1020	27.40	433	31.31	1933	30.13	1067	28.44	768	24.07	1818	27.95	1119	28.10	843	30.58	9001	28.39
42 months	949	25.50	417	30.15	1671	26.04	787	20.98	962	30.15	1856	28.53	1066	26.77	711	25.79	8419	26.55
Total	3722	100.00	1383	100.00	6416	100.00	3752	100.00	3191	100.00	6505	100.00	3982	100.00	2757	100.00	31,708	100.00
Children identified at high likelihood for autism by age bracket																		
12 months	4	5.19	2	7.14	10	19.23	18	18.18	11	23.91	7	6.86	7	12.50	3	5.17	62	11.97
18 months	20	25.97	4	14.29	6	11.54	24	24.24	13	28.26	13	12.75	15	26.79	12	20.69	107	20.66
24 months	27	35.06	12	42.86	10	19.23	43	43.43	10	21.74	32	31.37	16	28.57	8	13.79	158	30.50
42 months	24	31.17	10	35.71	21	40.38	14	14.14	12	26.09	43	42.16	17	30.36	27	46.55	168	32.43
42 months *	3	3.85	0	0.00	7	12.96	0	0.00	0	0.00	8	7.77	1	1.79	9	15.25	28	5.35
Total	78	100.00	28	100.00	54	100.00	99	100.00	46	100.00	103	100.00	56	100.00	59	100.00	523	100.00
Percentage	78	5.0	28	4.70	54	2.15	99	5.14	46	2.73	103	3.94	56	3.61	59	5.60	523	3.87
Research engagement																		
Attended	57	73.08	19	67.86	33	61.11	72	72.73	32	69.57	64	62.14	32	57.14	48	81.36	357	68.92
Declined	21	26.92	9	32.14	21	38.89	27	27.27	14	30.43	39	37.86	24	42.86	11	18.64	166	31.74

Notes: MCH; maternal and child health. *; children referred due to parental/caregiver and/or MCH nurse concerns.

**Table 3:** Child demographic and diagnostic characteristics according to maternal ethnicity.

	EME (n = 236)	MME (n = 15)	N-EME (n = 89)	Missing Data (n = 17)	Total (n = 357)	Difference ^1^
Demographic variables						
Sex, n (%)						
Female	54 (22.9)	1 (6.7)	20 (22.5)	3 (17.7)	78 (22.9)	Pearson chi2(3) = 2.3682 Pr = 0.500
Male	182(77.1)	14 (93.3)	69 (77.5)	14 (82.3)	279 (78.1)	
Total	236 (100.0)	15 (100.0)	89 (100.0)	17 (100.0)	357 (100.0)	
Country of birth, n (%)						
Australia	228 (96.6)	15 (100.0)	78 (92.9)	4 (23.5)	326 (95.9)	Pearson chi2(9) = 220.5623 Pr < 0.001
Other	6 (2.5)	0 (0.0)	5 (6.0)	0 (0.0)	11 (3.2)	
Missing data	2 (0.9)	0 (0.0)	1 (1.1)	13 (76.5)	3 (0.9)	
Total	236 (100.0)	15 (100.0)	89 (100.0)	17 (100.0)	357 (100.0)	
Language spoken at home, n (%)						
English only	210 (89.0)	13 (86.7)	28 (31.5)	2 (11.8)	253 (70.9)	Pearson chi2(9) = 375.3395 Pr < 0.001
Multilingual	22 (9.3)	2 (13.3)	51 (57.3)	1 (5.9)	76 (21.3)	
Non-English only	2 (0.8)	0 (0.0)	10 (11.2)	0 (0.0)	12 (3.4)	
Missing	2 (0.9)	0 (0.0)	0 (0.0)	14 (82.3)	16 (4.5)	
Total	236 (100.0)	15 (100.0)	89 (100.0)	17 (100.)	357 (100.0)	
Local government area, n (%)						
Banyule	39 (16.5)	0 (0.0)	13 (14.6)	5 (29.4)	57 (16.0)	Pearson chi2(24) = 65.7298 Pr < 0.001
Bayside	16 (6.8)	1 (6.7)	2 (2.3)	0 (0.0)	19 (5.3)	
Boroondara	22 (9.3)	3 (20.0)	5 (5.6)	2 (11.8)	32 (8.9)	
Hume	35 (14.8)	4 (26.7)	33 (37.1)	0 (0.0)	72 (20.2)	
Kingston	27 (11.4)	1 (6.7)	4 (4.5)	0 (0.0)	32 (9.0)	
Knox	41 (17.4)	2 (13.3)	19 (21.4)	2 (11.8)	64 (17.9)	
Moonee Valley	20 (8.5)	2 (13.3)	8 (9.0)	2 (11.8)	32 (8.9)	
Nillumbik	37 (15.4)	2 (13.3)	5 (5.6)	5 (29.4)	48 (13.5)	
Missing	0 (0.0)	0 (0.0)	0 (0.0)	1 (5.9)	1 (0.3)	
Total	236 (100.0)	15 (100.0)	89 (100.0)	17 (100.0)	357 (100.0)	
SEIFA- IRSAD Decile, n (%)						
Low	35 (14.8)	4 (26.7)	33 (37.1)	0 (0.0)	72 (21.3)	Pearson chi2(6) = 273.1901 Pr < 0.001
High	199 (84.3)	11 (73.3)	56 (62.9)	3 (17.7)	266 (78.7)	
Missing	2 (0.8)	0 (0.0)	0 (0.0)	14 (82.3)	16 (4.5)	
Total	236 (100.0)	15 (100.0)	89 (100.0)	17 (100.0)	357 (100.0)	
Maternal education (highest level completed), n (%)						
Secondary and primary education	43 (18.2)	3 (20.0)	11 (12.4)	0 (0.0)	57 (15.9)	Pearson chi2(6) = 15.2969 Pr = 0.018
Diploma and vocational education	65 (27.5)	2 (13.3)	10 (11.2)	1 (5.9)	78 (21.9)	
University	124 (52.5)	10 (66.7)	66 (72.6)	2 (11.8)	202 (56.6)	
Missing	4 (1.7)	0 (0.0)	2 (2.3)	14 (82.3)	20 (5.6)	
Total	236 (100.0)	15 (100.0)	89 (100.0)	17 (100.0)	357 (100.0)	
Current employment status, n (%)						
Employed	108 (45.7)	6 (40.0)	46 (51.7)	0 (0.0)	160 (47.1)	Pearson chi2(9) = 291.4910 Pr < 0.001
Unemployed	120 (50.9)	8 (53.3)	38 (42.7)	0 (0.0)	166 (48.8)	
Other	8 (3.4)	1 (6.7)	1 (1.1)	0 (0.0)	10 (2.9)	
Missing	0 (0.0)	0 (0.0)	4 (4.5)	17 (100.0)	21 (5.9)	
Total	236 (100.0)	15 (100.0)	89 (100.0)	17 (100.0)	357 (100.0)	
Annual family income (in Australian dollars), n (%)						
Low	22 (9.3)	1 (6.7)	8 (9.0)	1 (5.9)	32 (9.0)	Pearson chi2(9) = 42.2220 Pr < 0.001
Medium	125 (53.0)	8 (53.3)	52 (58.4)	1 (5.9)	186 (52.1)	
High	36 (15.3)	4 (26.7)	8 (9.0)	0 (0.0)	48 (13.4)	
Missing *	53 (22.5)	2 (13.3)	21 (23.6)	15 (88.2)	91 (25.5)	
Total	236 (100.0)	15 (100.0)	89 (100.0)	17 (100.0)	357 (100.0)	
Clinical characteristics						
Overall diagnosis, n (%)						
Autism	177 (75.0)	7 (46.7)	73 (82.0)	11 (64.7)	268 (75.1)	Pearson chi2(3) = 9.6873 Pr = 0.021
DD/LD	59 (25.0)	8 (53.3)	16 (18.0)	6 (35.3)	89 (24.9)	
Total	236 (100.0)	15 (100.0)	89 (100.0)	17 (100.0)	357 (100.0)	
Autism prevalence in each group						
	75. 0%	46.7%	82.0%	64.7%	75.1%	Pearson chi2(3) = 9.7411 Pr = 0.021

Note: DD/LD; developmental delay/language delay. IRSAD; Index for Relative Socioeconomic Advantage and Disadvantage. EME; European maternal ethnicity, MME; Mixed maternal ethnicity, N-EME; Non-European maternal ethnicity. SEIFA; Socioeconomic Index for Areas (SEIFA). Maternal employment status: employed (working full-time, working part-time, on maternity/paternity leave), unemployed (unemployed, unable to work, student, home duties), and other (self-employed, causal work, was part-time now casual). Maternal education: secondary and primary education (completed secondary education, some secondary education, completed primary education), diploma and vocational education (diploma to certificate I), and degree-level education (university education; advanced diploma to Ph.D.). Annual family income (in AUD): low (<$650 per week), medium ($651–2999 per week), and high (>$3000 per week) or missing (missing or did not want to answer) * *p* value < 0.05, ^1^ Differences explored using Pearson chi-square.

**Table 4:** Multinomial logistic regression analysis of the relative risk ratio of autism diagnosis compared to developmental and/or language delay (DD/LD) diagnosis of children from non-European and mixed maternal ethnicities compared to European maternal ethnicity.

Overall Diagnosis	EME (n = 241)			MME (n = 15)			N-EME (n = 84)		
	n	cRRR	aRRR *	n	cRRR	aRRR *	n	cRRR	aRRR *
		(95% CI)	(95% CI)		(95% CI)	(95% CI)		(95% CI)	(95% CI)
		*p* value	*p* value		*p* value	*p* value		*p* value	*p* value
DD/LD	Baseline								
Autism	241	Ref	Ref	15	0.29	0.26	84	1.52	1.33
					(0.10, 0.83)	(0.09, 0.78),		(0.81, 2.86)	(0.60, 2.92),
					*p* = 0.022	*p* = 0.017		*p* = 0.189	*p* = 0.480

Notes: aRRR; adjusted relative risk ratio, cRRR; crude relative risk ratio. DD/LD; developmental and/or language delay. Ref; reference level = 1.00. RRR; relative risk ratio. * Adjusted for maternal education (baseline secondary and lower compared to diploma and degree holders), languages spoken at home (baseline English only compared to non-English only and multilingualism), maternal employment status (current employed vs. unemployed status), and annual family income (baseline low compared to medium and high).

## Data Availability

The data presented in this study are available on request from the corresponding author. The data are not publicly available due to privacy and ethics.

## Supplementary Materials

The following supporting information can be downloaded at: https://www.mdpi.com/article/10.3390/brainsci13060903/s1, Table S1: Overall correlations between demographic and outcome measures; Table S2: Correlations between demographic and outcome measures children of EME; Table S3: Correlations between demographic and outcome measures for children of MME; Table S4: Correlations between demographic and outcome measures for children of N-EME.
