# Supplementary material for: Who Is Attending? The Role of Child Ethnicity and Maternal Demographics in Research Engagement and Early Identification of Autism
Source: Brain Sci. 2023 Jun 2;13(6):903. doi: 10.3390/brainsci13060903 (PMC10296669; doi:10.3390/brainsci13060903)
Supplement: Supplementary file 1 [file brainsci-13-00903-s001.zip › brainsci-2412265-supplementary.pdf]

**Table S1.** Overall correlations between demographic and outcome measures.

| | 2. Overall diagnosis | 3. LGA | 4. SEIFA | 5. Maternal education | 6. Maternal employment | 7. Language spoken at home | 8. Annual family income |
|---|---|---|---|---|---|---|---|
| 1. Child Ethnicity | 0.0530 | -0.0250 | -.2596** | .1370* | -0.0415 | .5614** | -0.0722 |
| 2. Overall Diagnosis | | -0.0349 | -.1260* | -0.0618 | .0582 | 0.0693 | -0.0550 |
| 3. LGA | | | .1253* | -0.0411 | 0.0158 | -0.0503 | -0.0848 |
| 4. SEIFA | | | | .1759** | -0.0808 | -.2575** | .1081* |
| 5. Maternal education | | | | | -.1637** | 0.0780 | .0.0979 |
| 6. Maternal employment | | | | | | -0.0455 | -.2353** |
| 7. Language spoken at home | | | | | | | 0.0294 |
| 8. Annual family income | | | | | | | |

Notes: LGA =Local Government Area, SEIFA; Socioeconomic Index for Areas.

**Correlation significant <0.01, * Correlation significant <0.05

**Table S2.** Correlations between demographic and outcome measures children of EME

| | 2. LGA | 3. SEIFA | 4. Maternal education | 5. Maternal employment | 6. Language spoken at home | 7. Annual family income |
|---|---|---|---|---|---|---|
| 1. Overall Diagnosis | -0.0138 | -0.0488 | -0.0678 | .1350* | 0.0287 | -0.0754 |
| 2. LGA | | 0.1040 | -0.1217 | 0.0640 | 0.0230 | -0.1058 |
| 3. SEIFA | | | .1464* | -0.0018 | -0.0307 | 0.0891 |
| 4. Maternal Education | | | | -0.0941 | 0.0457 | .2799** |
| 5. Maternal Employment | | | | | -0.0202 | -0.0760 |
| 6. Language spoken at home | | | | | | 0.0675 |
| 7. Annual family income | | | | | | |

Notes: LGA =Local Government Area, SEIFA; Socioeconomic Index for Areas.

**Correlation significant <0.01, * Correlation significant <0.05

**Table S3.** Correlations between demographic and outcome measures for children of MME

| | 2. LGA | 3. SEIFA | 4. Maternal education | 5. Maternal employment | 6. Language spoken at home | 7. Annual family income |
|---|---|---|---|---|---|---|
| 1. Overall Diagnosis | -0.1091 | -0.6447** | -0.3760 | .5229* | 0.4193 | -0.2334 |
| 2. LGA | | 0.2996 | 0.0206 | -0.0794 | -0.1949 | -0.1013 |
| 3. SEIFA | | | .5365* | -0.3371 | -.6504** | 0.1092 |
| 4. Maternal Education | | | | -0.0925 | -0.2272 | 0.0141 |
| 5. Maternal Employment | | | | | 0.2193 | -0.1667 |
| 6. Language spoken at home | | | | | | -0.1337 |
| 7. Annual family income | | | | | | |

Notes: LGA =Local Government Area, SEIFA; Socioeconomic Index for Areas.

**Correlation significant <0.01, * Correlation significant <0.05

**Table S4.** Correlations between demographic and outcome measures for children of N-EME

| | 2. LGA | 3. SEIFA | 4. Maternal education | 5. Maternal employment | 6. Language spoken at home | 7. Annual family income |
|---|---|---|---|---|---|---|
| 1. Overall Diagnosis | 0.0424 | -0.1841 | 0.1119 | -0.0541 | -0.0340 | -0.0787 |
| 2. LGA | | 0.1854 | 0.0952 | -0.1511 | -0.1446 | 0.0927 |
| 3. SEIFA | | | 0.3391** | -0.2169* | -.2316* | 0.0968 |
| 4. Maternal Education | | | | -.0753 | -0.0616 | 0.2483* |
| 5. Maternal Employment | | | | | 0.0854 | -.0929 |
| 6. Language spoken at home | | | | | | 0.1816 |
| 7. Annual family income | | | | | | |

Notes: LGA =Local Government Area, SEIFA; Socioeconomic Index for Areas.
**Correlation significant <0.01, * Correlation significant <0.05
